# Plant background, not *Bt* proteins, drives *Daphnia magna* life table responses: a multi-tissue chronic study with multi-Cry *Bt* maize

**DOI:** 10.3389/fpls.2026.1852457

**Published:** 2026-06-23

**Authors:** Yi Chen, Hang Tang, Shulin Ma, Xiaotian Lin, Jingyuan Guo, Ruizong Jia, Yishi Duan, Yan Yang

**Affiliations:** 1 Sanya Research Institute & Institute of Tropical Bioscience and Biotechnology, Chinese Academy of Tropical Agricultural Sciences, Sanya, Hainan, China; 2 Key Laboratory of Genetics and Germplasm Innovation of Tropical Special Forest Trees and Ornamental Plants, Ministry of Education & School of Tropical Agriculture and Forestry (School of Agricultural and Rural Affairs, School of Rural Revitalization), Hainan University, Hainan, China

**Keywords:** *Bt* maize, Cry proteins, *Daphnia magna*, environmental risk assessment, natural variation baseline, plant genetic background

## Abstract

**Introduction:**

The environmental safety of genetically engineered (GE) crops expressing insecticidal proteins remains debated, partly because observed biological effects are often difficult to distinguish from natural variation in plant genetic background.

**Methods:**

Here, we conducted a 21-day chronic feeding experiment to assess the effects of two *Bt* maize lines expressing three insecticidal proteins on the aquatic model organism *Daphnia magna*. Individuals were exposed to four ecologically relevant maize materials (pollen, leaves, flour, and straw leachate), alongside near-isogenic non-engineered controls and eight conventional maize varieties used to establish a natural variation baseline.

**Results:**

Protein concentrations varied substantially among tissues and between maize lines. However, life-table responses of *D. magna*, including survival, reproduction, and growth, were more strongly influenced by plant genetic background than by the presence of insecticidal proteins. Importantly, nearly all response values observed under exposure to *Bt* maize fell within the natural variation range defined by conventional varieties.

**Discussion:**

These results indicate that the tested maize lines do not cause biologically meaningful effects beyond the variability inherent to conventional crops. More broadly, this study demonstrates that incorporating natural variation baselines provides a robust and generalizable framework for distinguishing trait-related effects from background variability, thereby improving the scientific basis of environmental risk assessment for GE crops.

## 1 Introduction

*Bacillus thuringiensis* (*Bt*) maize, engineered to express insecticidal Cry proteins, has been widely adopted globally due to its enhanced insect resistance and reduced reliance on chemical insecticides (Cheng et al., 2024). However, the potential release of Cry proteins via pollen drift, plant residue decomposition, and leachate diffusion raises concerns about unintended effects on non-target organisms in aquatic ecosystems (Carstens et al., 2012; Chen et al., 2013), underscoring the need for thorough environmental risk assessment. *Daphnia magna*, a keystone species in aquatic food webs and a standard model in ecotoxicology, offers a sensitive system for such assessments (ASTM (American Society for Testing and Materials), 2005; ISO (International Organization for Standardization), 2012; OECD (Organization for Economic Co-operation and Development), 2004, 2012). Its life-table parameters, including survival, fecundity, and development time, reflect population-level responses to stressors and provide robust data for ecological risk evaluation (ASTM (American Society for Testing and Materials), 2005; ISO (International Organization for Standardization), 2012; OECD (Organization for Economic Co-operation and Development), 2004, 2012).

Previous studies have evaluated the effects of different *Bt* maize tissues on *D. magna*, yielding inconsistent results that highlight the complexity of such assessments. Exposure to pollen containing Cry1F or Cry1Ab proteins showed no adverse effects in short-term (48 h) tests (Mendelson et al., 2003). In contrast, pulverized leaves from Cry1Ab-expressing maize were reported to reduce growth and fecundity (Holderbaum et al., 2015). Studies on maize flour have presented clear contradictions: some found that Cry1Ab *Bt* maize flour reduced survival and reproduction compared to non-*Bt* isolines (Bøhn et al., 2008, 2010), while others detected no significant differences between *Bt* and non-*Bt* flour (Zhang et al., 2018, 2019). For straw, leachates have been shown to affect somatic growth and reproduction, identifying it as a potential exposure pathway (Tian et al., 2017).

The interpretability of these findings is constrained by two overarching limitations. First, key experimental conditions, such as exposure duration or photoperiod, often lack ecological realism or may introduce confounding stressors (e.g., Mendelson et al., 2003; Holderbaum et al., 2015), limiting their environmental relevance. Second, and more critically, the potential confounding effect of plant background is frequently overlooked. Differences in genetic plant background can independently influence plant tissue properties and organismal responses (Barbary et al., 2014). Many of the cited studies either did not specify the genetic relationship between compared materials (Bøhn et al., 2008, 2010) or provided insufficient details on cultivation and background genetics (Zhang et al., 2018, 2019), making it difficult to attribute observed effects unequivocally to the *Bt* trait rather than to inherent plant variations. Chen et al. (2021a) conducted a systematic comparison of *D. magna* performance fed with flour, leaves, and pollen from various maize lines, highlighting the inherent variation among non-*Bt* materials and underscoring the necessity of including multiple conventional varieties as reference points. Furthermore, Chen et al. (2021b) explicitly addressed the methodological hurdles in non-target feeding studies with stacked *Bt* maize, emphasizing that inconsistent results often arise from unstandardized materials and uncontrolled confounding factors. Without accounting for natural variation among conventional maize varieties, observed effects may be mistakenly attributed to the *Bt* trait rather than to plant background genetic differences.

Therefore, to address these gaps, this study investigated two *Bt* maize lines (from the same company) expressing three identical Cry proteins (Cry1Ab, Cry2Ab, Cry1F), along with their non-*Bt* near-isolines. We assessed the influence of four key tissues: pollen, leaves powder, maize flour powder, and straw soaking solution, on the life-table parameters of *D. magna*. To isolate the effect of the *Bt* trait from that of the plant background, we additionally included eight conventional maize varieties. The natural variation in life-table responses across ten maize varieties (two non-*Bt* controls plus eight conventional lines) was quantified to provide a reference baseline. The specific objectives were to:

1. compare the effects of multi-tissues from two Cry-protein-identical *Bt* maize lines on *D. magna* life-table parameters;
2. quantify the natural variation of these parameters across conventional maize varieties to distinguish plant background effects from *Bt*-specific impacts; and
3. provide a more scientifically robust framework for the environmental risk assessment of *Bt* maize.

This work aims to enhance the standardization of non-target risk assessment for genetically engineered (GE) crops and to support evidence based decision making for the commercial use and environmental management of *Bt* maize.

## 2 Materials and methods

### 2.1 Plant materials

To establish a baseline for natural variation in *D. magna* responses, eight conventional maize varieties with diverse genetic backgrounds were selected: Lianchuang 839 (LC839), Ludan 9191 (LD9191), Longping 722 (LP722), Longtian 5173 (LT5173), Longyuan 916 (LY916), Liangyu 99 (LY99), Qinfeng 108 (QF108), Yudan 517 (YD517). In addition, two *Bt* maize lines (HY1LP and MY73LP, express the same three Cry proteins: Cry1Ab, Cry1F, Cry2Ab) and the corresponding non-*Bt* maize lines (HY1 and MY73) were used.

All maize lines were planted in June 2023 in Longping Biotechnology (Hainan) Co., Ltd. Transgenic Experimental Base (Henan, China; 35°14′37″N, 113°58′35″E). Maize plants were cultivated according to commonly used local agricultural practices. All maize materials (pollen, leaves, flour and straw) were obtained from Longping Biotechnology (Hainan) Co., Ltd. (Hainan, China), and were prepared and stored according to Chen et al. (2021a). Briefly, the collected pollen of each maize line was poured through a 200 μm gauze and stored in screw-cap glass tubes at -70 °C. The leaves of each maize line were cut into pieces, lyophilized, ground, sieved through a 75 μm metal sieve and stored at -70 °C. Maize flour of each line was produced from the original batch of (untreated) seeds, maize grains were ground, sieved through a 75 μm metal sieve and stored at -70 °C. Maize straw of each line (cut into 1 cm pieces) from 20 cm above the soil surface were collected, pooled, and stored at -70 °C.

For the feeding assays, the sieved maize materials (pollen, leaves, flour) were suspended in Aachener Daphnien Medium (ADAM) (Klüttgen et al., 1994, medium composition modified after Ebert et al., 1998) at 3 mg/mL, and stored in 2 ml aliquots at -20 °C. Straw soaking solution were prepared by immersing 1 cm maize straw pieces in 3 L conical flasks with 1500 mL ADAM medium (0.4 g/L) at 20 °C for 24 h, followed by centrifugation (15,700×g for 5 min at 4 °C), and supernatants filter-sterilized via a 0.22-µm Millipore filter (Chen et al., 2018b).

### 2.2 Algae and *D. magna*

Algae *Chlorella pyrenoidosa* (Chlorellales: Oocystaceae), designated as the optimal dietary source for *Daphnia magna*, and a monoclonal strain of *D. magna* were kindly provided by Dr. Wang Jiamei (College of Marine and Bioengineering, Yancheng Institute of Technology, China). *D. magna* cultures were maintained in ADAM medium within a climate-controlled incubator, with conditions set at 25 °C, 70% relative humidity (RH), and a 16:8 h light:dark photoperiod. The medium was formulated and agitated at ambient temperature for a minimum of 12 hours prior to use. Every 14 days, *D. magna* individuals were subcultured into fresh medium using Pasteur pipettes. Cultured *D. magna* displayed no indicators of stress, including the absence of males or ephippia, no body discoloration, and low mortality rates. *D. magna* were fed daily a diet of algae at a concentration of 3×10^6^ cells/mL.

### 2.3 Exposure of *D. magna* to maize materials

Neonates (within 6–24 h of hatching) from the culture were kept individually in 100 mL glass beakers containing 50 mL ADAM medium (pollen, leaves and flour treatments) or straw soaking solution (straw treatments) following test procedures of ISO 6341 and OECD 211 (ISO (International Organization for Standardization), 2012; OECD (Organization for Economic Co-operation and Development), 2012). For pollen, leaves, and flour treatments, individuals were fed 100 μL of the 3 mg/mL relatively suspension of maize materials (approx. 0.15 mg C) daily. For straw treatments, the straw leachate alone contains no particulate matter and cannot be directly ingested by *D. magna*. Therefore, animals were fed 10 million *C. pyrenoidosa* cells (approx. 0.15 mg C) daily as a food source, while the leachate served as the vehicle for potential Cry protein exposure. This approach reflects the field scenario where *D. magn*a consume algae and other microorganisms that may have adsorbed Cry proteins leached from straw residues (Chen et al., 2018b; Tian et al., 2017). In contrast, pollen, leaves, and flour are solid particulate materials that *D. magna* can directly filter and ingest; hence, no additional algae were needed. The feeding ration was based on organic carbon content and the recommended feeding ration, per *D. magna* per day is between 0.1 and 0.2 mg C (OECD (Organization for Economic Co-operation and Development), 2012; Hart et al., 2007; Meissle et al., 2011; Chen et al., 2021a).

For each combination of the four maize material treatments (pollen, leaves, flour, straw) and twelve maize lines described before in 2.1 (eight conventional lines, two *Bt* maize lines, and two corresponding non-*Bt* maize lines), ten individuals were tested. The sample size of n = 10 per treatment follows the OECD 211 guideline (OECD (Organization for Economic Co-operation and Development), 2012) for *D. magna* reproduction tests and is consistent with previous studies (Chen et al., 2021a, 2021b), thus the total number of *D. magna* in this experiment was 480. The experiment was conducted in a climate chamber (20 °C, 70% RH) under a 16 h light/8 h dark cycle. Food (pollen, leaves, flour, algae) was provided daily throughout the experiment. Every third day, *D. magna* were moved to a new beaker with ADAM medium to ensure that the medium quality remained stable. The following parameters were recorded daily: number of surviving *D. magna*, molts, and released offspring. All offspring were removed after counting. The experiment was terminated after 21 days, at which time body size (body length and body width) and body weight were measured for each surviving adult. Pictures were taken by motorized stereomicroscope (SZX16, Olympus, Tokyo, Japan) and were subsequently measured with ImageJ (ImageJ 1.54g, Java 1.8.0_345, National Institutes of Health, USA). Intact live individuals were rinsed with fresh ADAM medium, blotted dry using absorbent paper towels, carefully transferred into 2 mL centrifuge tubes, and subsequently weighed using an electronic analytical balance (JY20002, Shanghai Hengping Instrument Co., Ltd., Shanghai, China). All individuals were then stored at −70 °C for subsequent determination of Cry protein content using ELISA (see below).

### 2.4 Medium quality analyses

The qualities of the ADAM medium of all treatments or straw soaking solution quality parameters (pH and dissolved oxygen concentration, DOC) were measured at multiple time points using a pH meter (PE28, Mettler Toledo Instruments Co., Ltd., Shanghai, China) and a multi-parameter meter (SX736, Shanghai Sanxin Instrument Factory, Shanghai, China). The sampling timeline, corresponding operations, and measured parameters are summarized in **Table 1**. All values remained within the ranges recommended by OECD 211 (OECD (Organization for Economic Co-operation and Development), 2012).

**Table 1:** The qualities of the ADAM medium of all treatments or straw soaking solution quality parameters (pH and dissolved oxygen concentration, DOC) sampling time points.

Time point	Description
W0	Pure ADAM medium or straw soaking solution (initial)
W1	After adding food (pollen/leaves/flour) or algae (to straw)
W2	24 h after adding food (with one *D. magna*)
W3	W2 plus one additional food dose for 1 day
W4	W3 after another 24 h
W5	W4 plus one additional food dose for 1 day
W6	W5 after another 24 h

### 2.5 Determination of Cry protein content by ELISA

Cry protein (Cry1Ab, Cry1F, Cry2Ab) contents in maize materials (pollen, leaves, flour and straw) and in *D. magna* were analyzed using enzyme-linked immunosorbent assays (ELISA) with corresponding Cry protein detection kit (Fankew, China). Four replicates of maize materials and three replicates of *D. magna* were suspended in 650 μL of PBST extraction buffer in 1.5 mL centrifuge tubes. The samples were fully ground using an electric grinding rod. All samples were centrifuged at 13,000 × *g* for 5 min at 4 °C. The supernatants (600 μL each) were then collected in new tubes. ELISA was performed after centrifugation according to the manufacturer’s instructions. Optical density was read at 450 nm with a plate reader (SpectraMax^®^ ABS Plus, Molecular Devices, LLC, California, United States).

### 2.6 Data analysis

Data were analyzed using R, version 4.5.2 (The R Foundation for Statistical Computing, Vienna, Austria). All data are presented as mean ± standard error (SE), unless otherwise indicated. The measured parameters of *D. magna* were analyzed separately for pollen, leaves, flour and straw.

Survival probability was analyzed by Kaplan-Meier estimates and log-rank tests (survival package). HY1 and MY73 represent two distinct genetic backgrounds of maize. Parameters (moltings to first offspring, first offspring time, individuals in the first clutch, total clutches) were analyzed with generalized linear mixed effects models (GLMER) with plant background (two levels: HY1, MY73) and *Bt* (Bt^+^, Bt^-^) as fixed factors, and each individuals as random factor (lme4 package) according to Chen et al. (2021b). Parameters (total offspring and offspring per clutch) were analyzed with negative binomial generalized linear model (NB-GLM) with plant background (HY1, MY73) and *Bt* (Bt^+^, Bt^-^) as fixed factors. Other parameters (body weight, body length and body width) were analyzed with linear model (LM) with plant background (HY1, MY73) and *Bt* (Bt^+^, Bt^-^) as fixed factors. Differences were considered significant at *p* < 0.05. When interactions between the factors plant background and *Bt* were significant, separate analyses for both factors were conducted.

The variation range (VR) was calculated from the ten non-*Bt* lines (i.e., LC839, LD9191, LP722, LT5173, LY916, LY99, QF108, YD517, HY1, MY73) tested in parallel with the two *Bt* lines. Since the data were discrete proportional data, the 95% confidence interval (95CI) calculated by the conventional normal approximation method yielded negative values. Therefore, we used the nonparametric quantile method to compute the interval, the valid data were sorted in ascending order, and the 5th percentile (P5) and 95th percentile (P95) were selected, the interval [P5, P95] was regarded as the 95% empirical interval, which was defined as the VR.

For ELISA analyses, standard curves were constructed using a single rectangular hyperbola model. Concentrations of each Cry protein in the samples were derived from the respective standard curves. To determine the ELISA limit of detection (LOD) for each Cry protein, the standard deviation of blank measurements across two ELISA plates was calculated. The LOD was defined as three times this standard deviation, and the corresponding LOD concentrations (µg/g) for each plate and sample were computed via the respective standard curves, following the method described by Chen et al. (2021b). ELISA data were analyzed using median and 95CIs, which are robust for small sample sizes and non-normal distributions. Differences were considered significant for non-overlapping 95CIs.

## 3 Results

### 3.1 Medium quality

All values for the water quality (pH, DOC) were within the range demanded in OECD211 (OECD (Organization for Economic Co-operation and Development), 2012), i.e., pH 6 - 9, DOC > 3 mg/L (**Supplementary Table 1**). Briefly, all pH values of the ADAM medium were between 7.02 and 7.82, and all DOC values were between 6.64 mg/L and 9.11 mg/L, confirming that medium conditions did not confound the experimental results.

### 3.2 Cry protein concentrations in maize materials and *D. magna*

Cry protein concentrations varied markedly among maize materials (pollen, leaves, flour, straw) and between the two *Bt* lines (HY1LP, MY73LP) (**Table 2**). Total Cry protein was highest in leaves (HY1LP: 30.7 μg/g; MY73LP: 13.7 μg/g), intermediate in pollen (HY1LP: 3.4 μg/g; MY73LP: 5.6 μg/g) and straw (HY1LP: 2.8 μg/g; MY73LP: 7.1 μg/g), and lowest in flour (HY1LP: 4.2 μg/g; MY73LP: 2.5 μg/g). The total concentration in leaves was approximately 5 to 7 times higher than that in flour. Among the tested proteins, the concentration of Cry1F was the lowest in pollen, and that of Cry1Ab was the minimum in leaves. Notably, Cry2Ab displayed the highest concentration in flour, whereas its concentration was the lowest in straw. Cry protein concentrations further differed between the two *Bt* maize lines. Specifically, MY73LP pollen had significantly higher Cry1Ab levels than HY1LP pollen, whereas MY73LP leaves showed markedly lower concentrations of all Cry proteins relative to HY1LP leaves. For Cry1F, MY73LP flour contained significantly lower concentrations than HY1LP flour. In contrast, MY73LP straw exhibited significantly higher concentrations of both Cry1Ab and Cry1F than HY1LP straw. No Cry proteins were detected in HY1 or MY73 maize materials.

**Table 2:** Cry protein concentrations (μg/g dry weight) in pollen, leaves, flour and straw from two *Bt* maize hybrids.

Cry protein	Pollen		Leaves		Flour		Straw	
	HY1LP	MY73LP	HY1LP	MY73LP	HY1LP	MY73LP	HY1LP	MY73LP
Cry1Ab	1.0 (0.7; 1.4)	2.8 (2.3; 3.4)	9.1 (5.6; 9.4)	4.0 (3.2; 4.8)	0.9 (0.5; 1.2)	0.8 (0.7; 0.9)	1.2 (0.8; 2.2)	3.4 (2.6; 3.8)
Cry1F	0.7 (0.6; 1.0)	0.9 (0.7; 1.1)	12.4 (10.3; 14.9)	4.0 (2.8; 5.4)	1.3 (1.1; 1.4)	0.3 (0.3; 0.5)	1.3 (1.0; 1.6)	3.0 (2.3; 4.3)
Cry2Ab	1.7 (0.6; 3.8)	1.9 (1.4; 2.3)	9.2 (7.4; 13.4)	5.7 (5.2; 6.7)	2.0 (1.8; 2.5)	1.4 (0.9; 1.8)	0.3 (0.2; 0.6)	0.7 (0.5; 0.8)
Total	3.4	5.6	30.7	13.7	4.2	2.5	2.8	7.1

Data are presented as median ± 95CI for each hybrid (n = 4 independent biological samples). Non−overlapping 95% confidence intervals indicate a statistically significant difference between groups.

In *D. magna* fed *Bt* maize materials, Cry1Ab was consistently below the LOD across all treatments. Cry1F and Cry2Ab were detected, with median concentrations ranging from 0.09 to 0.33 μg/g for Cry1F and 0.11 to 0.70 μg/g for Cry2Ab (**Table 3**). Total Cry protein accumulation in *D. magna* was highest in the flour treatment of MY73LP (1.03 μg/g) and lowest in the straw treatment of HY1LP (0.20 μg/g). Cry protein concentrations did not differ significantly between the two *Bt* maize lines (**Table 3**). No Cry proteins were detected in *D. magna* individuals fed with HY1 or MY73 maize materials.

**Table 3:** Cry protein concentrations (μg/g dry weight) in *Daphnia magna* fed with maize materials pollen, leaves, flour and straw from two *Bt* maize hybrids.

Cry protein	Pollen		Leaves		Flour		Straw	
	HY1LP	MY73LP	HY1LP	MY73LP	HY1LP	MY73LP	HY1LP	MY73LP
Cry1Ab	< 0.105	< 0.103	< 0.142	< 0.142	< 0.094	< 0.241	< 0.061	< 0.065
Cry1F	0.15 (0.06; 0.35)	0.14 (0.09; 0.18)	0.22 (0.17; 0.25)	0.19 (0.14; 0.28)	0.15 (0.10; 0.24)	0.33 (0.13; 0.58)	0.09 (0.03; 0.22)	0.09 (0.06; 0.12)
Cry2Ab	0.21 (0; 0.76)	0.27 (0.17; 0.34)	0.29 (0.15; 0.51)	0.31 (0.22; 0.38)	0.19 (0.12; 0.33)	0.70 (0.32; 1.10)	0.11 (0.08; 0.21)	0.25 (0.17; 0.29)
Total	0.36	0.41	0.51	0.50	0.34	1.03	0.20	0.34

Data are presented as median ± 95CI for each hybrid (n = 4 independent biological samples). Non−overlapping 95% confidence intervals indicate a statistically significant difference between groups.

### 3.3 Performance of *D. magna* on maize materials

*Daphnia magna* was fed pollen, pulverized leaves, exclusively flour or straw soaking solution containing algae (*Chlorella pyrenoidosa*) from two *Bt* maize lines (HY1LP and MY73LP), two non-*Bt* nearest comparator lines (HY1 and MY73, respectively), and eight unrelated non-*Bt* maize line (LC839, LD9191, LP722, LT5173, LY916, LY99, QF108, YD517) for 21 days. Life table parameters of *D. magna* fed *Bt* lines or their comparators were assessed statistically.

**Figure 1:**
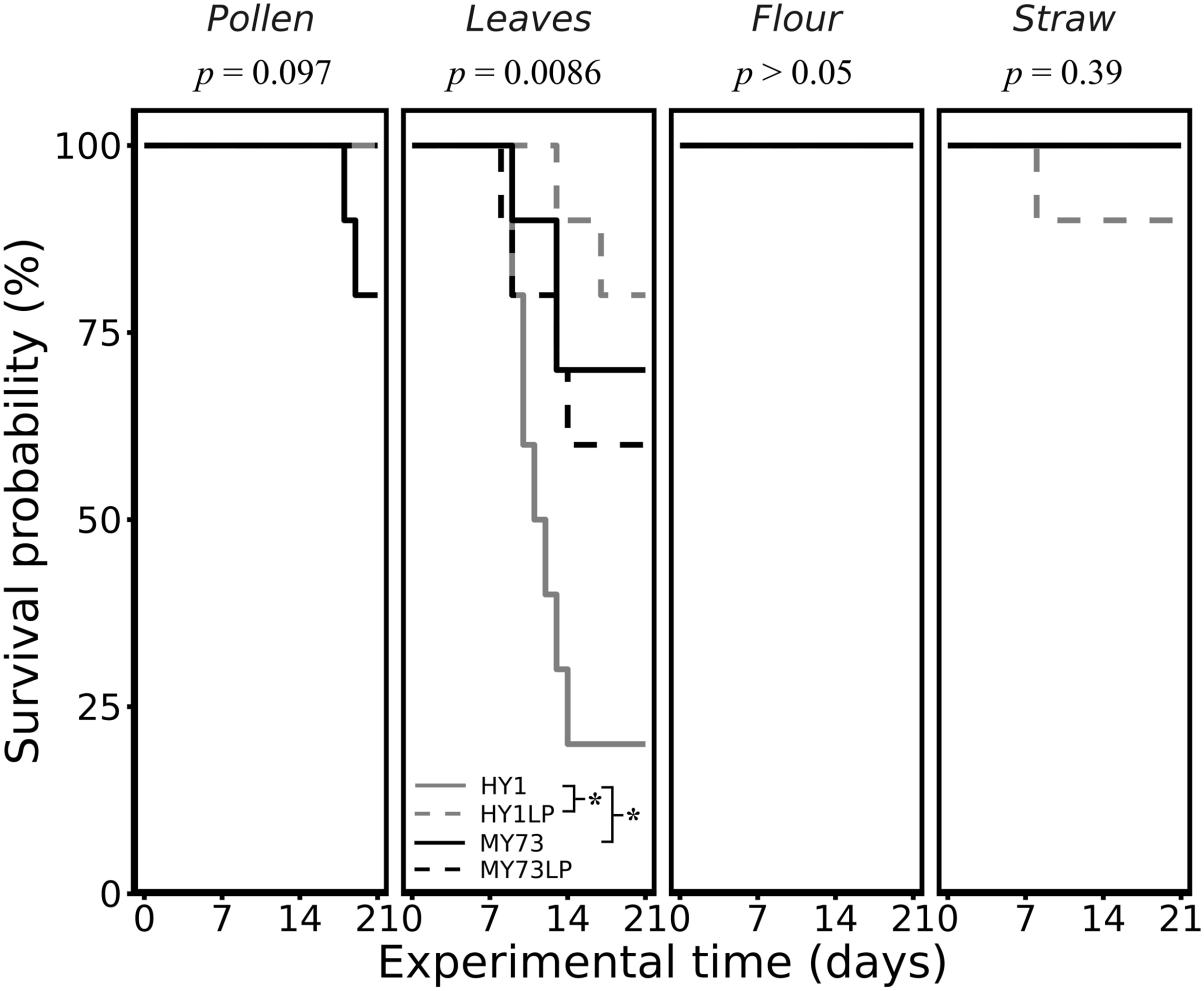
Survival of *Daphnia magna* fed pollen, leaves, flour or straw soaking solution containing algae (*Chlorella pyrenoidosa*) from four maize lines (HY1, HY1LP, MY73, MY73LP) (n = 10). Data were separately analyzed for each maize material using Kaplan-Meier estimates and log-rank tests. Pairwise comparisons were performed using log-rank tests with Bonferroni correction; only significant differences (*p* < 0.05) are marked with asterisks.

The survival probability of *D. magna* for maize leaves differed on different maize lines (Kaplan-Meier procedure and log-rank test: χ^2^ = 11.7, *p* = 0.0086). Survival probability was higher when *D. magna* fed HY1LP rather than HY1 (*Bt* effect: χ^2^ = 9.2, *p* = 0.002); when fed MY73 rather than HY1 (plant background effect: χ^2^ = 5.4, *p* = 0.02). For pollen, flour, and straw treatments, survival did not differ significantly among lines (**Figure 1**). Notably, the survival probability of *D. magna* for maize flour on different maize lines were all 100%.

**Figure 2:**
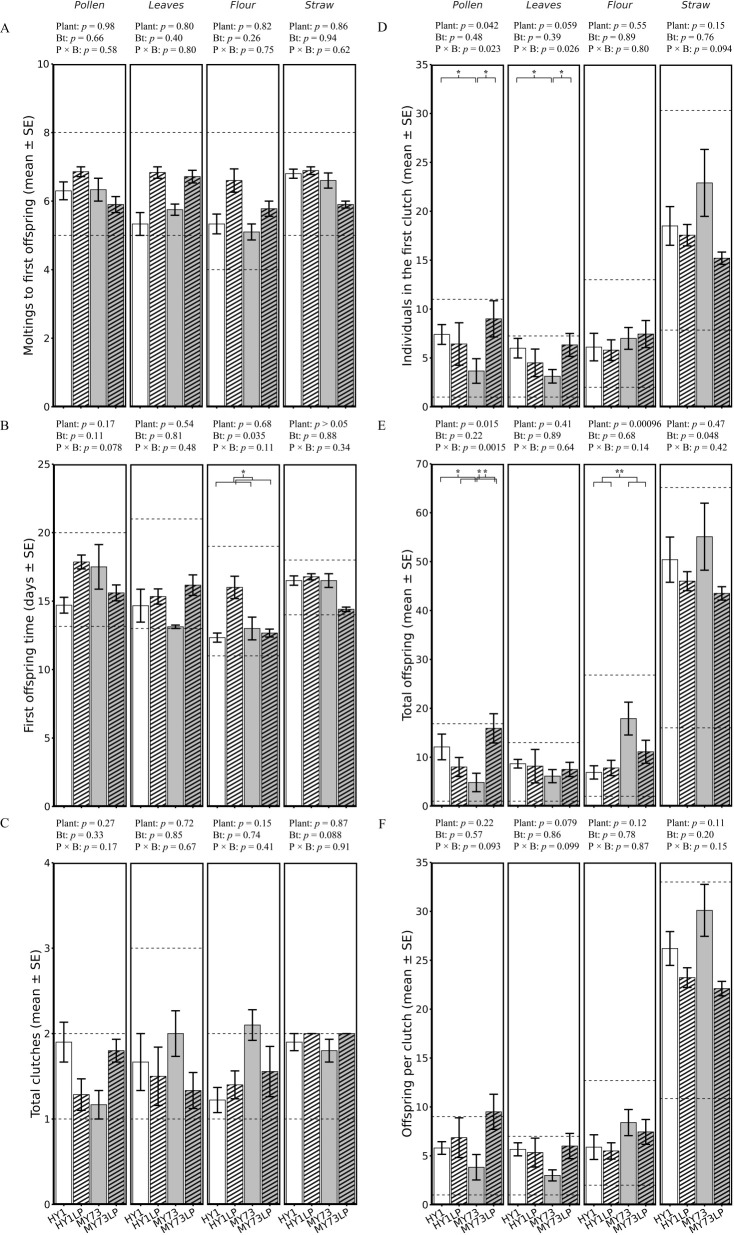
Number of molts to first offspring release **(A)**, time to first offspring release **(B)**, total number of clutches **(C)**, number of individuals in the first clutch **(D)**, total number of offspring **(E)** and number of offspring per clutch **(F)** of *Daphnia magna* fed pollen, leaves, flour or straw soaking solution containing algae (*Chlorella pyrenoidosa*) from four maize lines (HY1, HY1LP, MY73, MY73LP). Data were analyzed using GLMER **(A–D)** or NB-GLM **(E, F)** with plant background (HY1, MY73) and Bt (Bt+, Bt-) as fixed factors. Asterisks indicate significant differences (*p ≤ 0.05, **p ≤ 0.001) (n = 10). Dashed lines indicate the variation range (VR) from ten unrelated non-Bt maize lines (LC839, LD9191, LP722, LT5173, LY916, LY99, QF108, YD517, HY1, MY73).

The number of molts to first offspring release, total number of clutches and number of offspring per clutch produced by *D. magna* were not affected by the factors plant background or *Bt* for any of the maize materials (**Figures 2A, C, F**). For maize flour treatments, the time to first offspring release was significantly affected by *Bt* but not by plant background (**Figure 2B**). The number of offspring in the first clutch was significantly affected by both plant background and *Bt* for pollen and leaves treatments (**Figure 2D**), i.e., individuals produced less offspring in the first clutch if fed MY73 rather than HY1 pollen or leaves (plant background effect) or MY73LP pollen or leaves (*Bt* effect). There were no significant differences in this parameter for flour or straw treatments. Total number of offspring showed significant effects in pollen and flour treatments (**Figure 2E**). For pollen treatments, both plant background and *Bt* status were significant: *D. magna* fed MY73 had less total offspring than those fed HY1 (plant background effect) or MY73LP (*Bt* effect). In addition, *D. magna* fed MY73LP had more offspring than those fed HY1LP (plant background effect). For flour treatments, a strong plant background effect was observed: *D. magna* fed MY73 and MY73LP had more offspring than those fed HY1 and HY1LP, respectively (plant background effect).

**Figure 3:**
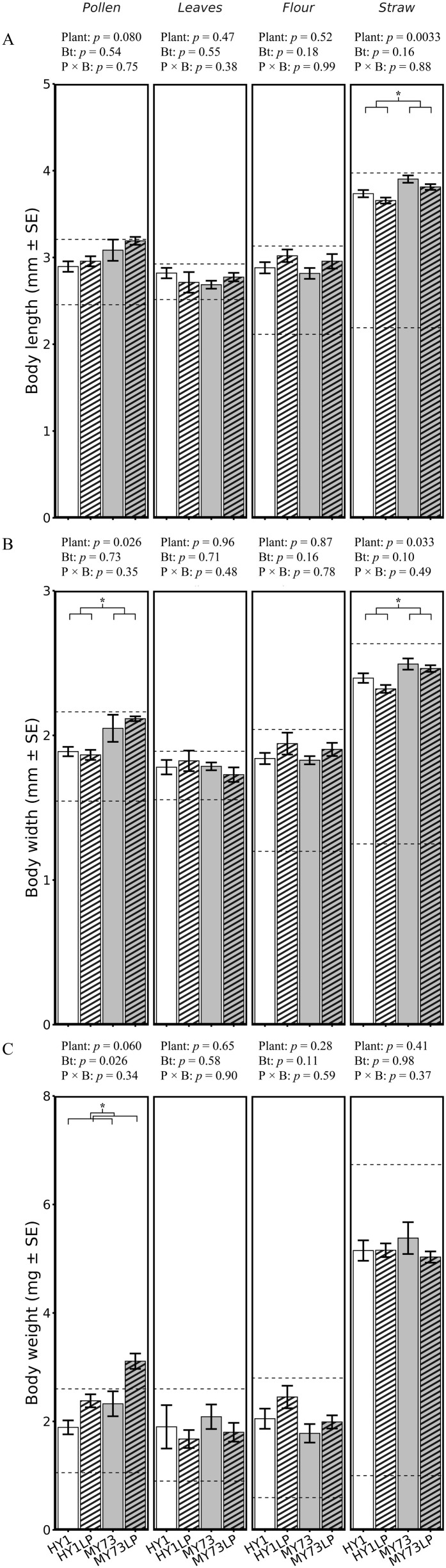
Body length **(A)**, body width **(B)** and body weight **(C)** of *Daphnia magna* fed pollen, leaves, flour or straw soaking solution containing algae (*Chlorella pyrenoidosa*) from four maize lines (HY1, HY1LP, MY73, MY73LP). Data were analyzed using LM with plant background (HY1, MY73) and Bt (Bt+, Bt-) as fixed factors. Asterisks indicate significant differences (*p ≤ 0.05) (n = 10). Dashed lines indicate the variation range (VR) from ten unrelated non-Bt maize lines (LC839, LD9191, LP722, LT5173, LY916, LY99, QF108, YD517, HY1, MY73).

For the body length (maize straw treatments) (**Figure 3A**) and body width (maize pollen and straw treatments) (**Figure 3B**) were significantly affected by plant background but not by *Bt*. The body weight was significantly affected by *Bt* but not by plant background for pollen treatments (**Figure 3C**). There were no significant differences in other parameters.

To evaluate how the mean values of various measured parameters align with the natural variation range of conventional maize lines, we computed a variation range (VR) based on the 5th percentile (P5) and 95th percentile (P95) of ten unrelated non-*Bt* maize inbred lines (LC839, LD9191, LP722, LT5173, LY916, LY99, QF108, YD517, HY1, MY73). When compared to this VR (dashed lines in **Figures 2**, **3**) most treatment means fell within the range, with the following exceptions: with MY73 flour, total number of clutches of *D. magna* was above the VR (**Figure 2C**); with MY73LP pollen, the number of offspring per clutch and body weight of *D. magna* were above the VR (**Figures 2F**, **3C**).

## 4 Discussion

This study systematically assessed the potential effects of two *Bt* maize lines, expressing identical Cry1Ab, Cry2Ab, and Cry1F proteins, on the keystone aquatic crustacean *D. magna*. By employing a multi-tissue exposure design (pollen, leaves, flour, straw leachates) and incorporating a panel of eight conventional maize varieties to establish a natural variation range (VR), we aimed to disentangle the effects of the *Bt* trait from those of the plant background effect. Our key findings reveal that: (1) Cry protein concentrations varied dramatically among maize materials and between the two *Bt* lines; (2) the life table responses of *D. magna* were more frequently and strongly influenced by the plant background effect than by the presence of Cry proteins; and (3) nearly all observed parameter means for *Bt*-exposed *D. magna* fell within the VR defined by ten non-*Bt* lines, indicating that any effects were within the spectrum of VR among conventional maize.

### 4.1 Tissue-specific exposure profiles and environmental implications

The ELISA results demonstrated a clear tissue-specific expression and/or leaching profile for the three Cry proteins, with concentrations highest in leaf powder and lowest in flour, differing by a factor of 5-7. This pattern aligns with known tissue-specific promoter activity driving transgene expression in leaves (Van Frankenhuyzen, 2013). Notably, the distribution of individual Cry proteins also varied by tissue (e.g., Cry2Ab highest in flour, lowest in straw), and significant differences existed between the two *Bt* lines (HY1LP and MY73LP) for specific protein-tissue combinations. This inter-line variability underscores that even with identical transgenes, the genetic plant background can modulate protein accumulation, a phenomenon consistent with previous observations that tissue- and hybrid-specific factors can influence the expression and effects of *Bt* proteins in non-target studies (Jensen et al., 2010; Holderbaum et al., 2015). From an environmental risk perspective, these findings highlight that exposure estimates for aquatic systems cannot be extrapolated from one tissue to another. Leaves and straw, likely entering waterways via edge-of-field runoff or residue decomposition, may represent more significant vectors for Cry proteins than pollen or grain, corroborating concerns raised about plant residue inputs (Tian et al., 2017; Carstens et al., 2012). Importantly, despite the high Cry protein concentrations in leaves and straw, we observed no correspondingly strong Bt effects in these treatments, further arguing against direct toxicity.

We further note that the internal Cry protein concentrations in *D. magna* varied among treatments (**Table 3**), and several mechanisms may explain the observed patterns. Cry proteins are known to degrade rapidly in aquatic environments; Griffiths et al. (2009) reported that approximately 60% of Cry1Ab leaches from maize leaves within the first hour of submergence, and Prihoda and Coats (2008) found half-lives of Cry3Bb1 in submerged plant tissue of less than 3 days. Under our experimental conditions with medium renewal every three days, Cry protein concentrations in the water phase likely decreased substantially between renewals, resulting in pulsed rather than continuous exposure. Consequently, numerous previous studies attempting to quantify Cry proteins in water have consistently reported levels below the limit of detection (Douville et al., 2007; Tank et al., 2010; Wang et al., 2013). For this reason, we did not include such measurements here. Instead, we confirmed that *D. magna* actively ingested the maize materials by direct observation under a stereomicroscope: the transparent gut of *D. magna* clearly showed colored maize particles, as also demonstrated in our previous study (Chen et al., 2021b). This visual verification, together with the detection of Cry1F and Cry2Ab in *D. magna* tissues (**Table 3**), confirms that exposure via ingestion occurred. Additionally, *D. magna* may differentially digest or excrete the three Cry proteins; the consistently undetectable levels of Cry1Ab suggest that this protein is either less stable in the gut or more efficiently cleared compared to Cry1F and Cry2Ab. Adsorption of Cry proteins to organic particles or container walls may also reduce bioavailability (Carstens et al., 2012). We acknowledge that direct measurement of Cry proteins in the exposure medium was not performed, which is a limitation, and future studies may include such measurements at multiple time points to further characterize exposure dynamics.

### 4.2 Disentangling plant background effects from *Bt*-specific effects

The most significant outcome of this study is the clear separation of plant background effects from potential *Bt* effects. For the majority of life table parameters, including molts to maturation, total clutches, and offspring per clutch, no significant *Bt* effect was detected across all four materials. In contrast, plant background had a significant influence on several key parameters, such as total offspring in flour treatments, body size in straw treatments, and first-clutch offspring in pollen and leaf treatments.

This prevalence of plant background effects strongly suggests that inherent differences in the nutritional composition or levels of secondary metabolites among maize inbred lines exert a greater influence on *D. magna* fitness than the addition of Cry proteins (Raymond et al., 2011; Ricroch and Damave, 2016). The few instances where a significant *Bt* effect was observed (e.g., time to first offspring in flour, body weight in pollen treatments) were often context-dependent and intertwined with background effects. For example, the reduced total offspring in MY73LP pollen compared to its non-*Bt* comparator MY73 could theoretically be a *Bt* effect, but the simultaneous reduction in MY73 (non-*Bt*) compared to HY1 (non-*Bt*) demonstrates a strong confounding plant background effect. This complexity underscores the risk of misattribution in studies using single, poorly matched comparators, potentially explaining inconsistencies in earlier literature (Bøhn et al., 2008). Our design, using both near isogenic and diverse conventional controls, effectively mitigates this confound and aligns with previous studies that identified plant background as a major source of variation in non-target organism responses to GE crops (Jensen et al., 2010; Swan et al., 2009).

### 4.3 The natural variation range as a decisive risk assessment tool

Incorporating the VR calculated from ten non-*Bt* lines provides a robust, quantitative benchmark for ecological relevance. In this study, we defined VR as the P5 to P95 interval of data pooled from ten conventional non-*Bt* maize lines (LC839, LD9191, LP722, LT5173, LY916, LY99, QF108, YD517, HY1, MY73). This approach captures natural variability at the individual level, reflecting the range within which healthy *D. magna* populations can be expected to perform when feeding on conventional maize materials. The fact that all but three parameter means from *Bt* treatments lay within this VR is compelling evidence for a lack of biologically significant adverse effects. The few exceptions (e.g., increased body weight with MY73LP pollen, increased clutches with MY73 flour) are marginal deviations and, crucially, are not indicative of a consistent, adverse *Bt* pattern. Some even suggest a potential positive physiological response.

This “baseline” approach is increasingly advocated for non-target risk assessment of GE crops, as it contextualizes any observed difference against the normal variability found in conventional agriculture (Romeis et al., 2013; Rauschen et al., 2009). An additional insight emerges from comparing the VR established in this study with that reported in our previous work (Chen et al., 2021a; b). In that study, we characterized the variation among five Swiss conventional maize varieties (Rheintaler, Tasty Sweet, ES-Eurojet, Planoxx, EXP 258) grown under greenhouse conditions, using a different statistical approach: the variation range was defined by the lowest lower 95CI and the highest upper 95CI across varieties (Chen et al., 2021a). This method reflects the range of variety-specific mean performance, whereas our current approach captures individual-level variability. Despite these methodological differences, the magnitude of variation was remarkably consistent across datasets. For example, in flour treatments, total offspring varied by a factor of approximately 2.1 among Swiss varieties (from 46.2 for Rheintaler to 97.9 for ES-Eurojet) (Chen et al., 2021a), while among the ten Chinese varieties, the P5 to P95 range spanned a factor of approximately 2.3. Similarly, for leaf treatments, total offspring varied by a factor of approximately 2.5 among Swiss varieties, while the Chinese dataset showed a comparable factor of approximately 2.7. Body length variation also showed consistency: Chen et al. (2021a) reported coefficients of variation of approximately 5% to 8% across Swiss varieties, while our study yielded a similar range across ten Chinese varieties (**Figure 3A**, dashed lines).

This consistency across geographically and genetically distinct maize germplasms, despite differences in cultivation conditions (greenhouse vs. field), statistical approaches (variety-level CI ranges vs. individual-level percentiles), and specific varieties, suggests that the observed plant background effects reflect a general biological phenomenon rather than a specific attribute of particular varieties or cultivation conditions. Importantly, it reinforces the validity of using a baseline of conventional varieties to contextualize the responses of GE crops. Our results operationalize this concept for *D. magna* and multi-tissue exposure, demonstrating its utility in concluding no substantial additional risk from the tested *Bt* maize lines.

### 4.4 Reconciling with previous studies and implications for future ecological risk assessment

The minimal differences observed between *Bt* and non−*Bt* treatments in our study contrast with some previous reports of adverse effects of *Bt* maize on *D. magn*a (Bøhn et al., 2008, 2010; Holderbaum et al., 2015). A closer examination of experimental designs reveals key differences that likely explain these discrepancies. First, studies reporting adverse effects typically used only a single *Bt* line compared with a single non−*Bt* control, often without confirming the genetic relatedness of the two lines or accounting for plant background variation. In contrast, our study included two *Bt* lines with distinct genetic backgrounds, each paired with its own near−isogenic control, and further incorporated eight additional conventional varieties to establish a natural variation baseline. Second, the type and source of maize material differ substantially. Bøhn et al. (2008, 2010) used maize flour produced from field−grown grains, where cultivation conditions (location, year, management) likely differed between the *Bt* and non−*Bt* lines, introducing confounding nutritional differences. Holderbaum et al. (2015) used maize leaf powder from plants grown under controlled conditions but still compared only one *Bt* line with one non−*Bt* isoline. In our study, we tested four maize materials (pollen, leaves, flour, straw) and found that effects were most pronounced in flour, material most susceptible to variation in production conditions, whereas no consistent *Bt* effects were observed in leaves or pollen produced from plants grown concurrently under identical conditions. Third, most previous studies did not establish a baseline of natural variation among conventional maize lines, making it difficult to judge whether statistically significant differences between *Bt* and non−*Bt* lines are biologically meaningful. When we applied our VR framework to the data from Holderbaum et al. (2015) (as discussed in Chen et al., 2021b), the reported effects fell within or near the natural variation range, suggesting they may not represent true hazards. Taken together, these comparisons demonstrate that our multi−background, multi−tissue, baseline−inclusive design provides a more robust framework for distinguishing *Bt*−specific effects from plant background noise.

This study offers two major methodological advancements for the ERA of transgenic crops. First, it validates the necessity of a multi-tiered control strategy, that is combining near isogenic lines with a diverse set of conventional varieties, to conclusively isolate trait effects from plant background noise. Regulatory testing should move beyond the simple “one Bt vs. one isoline” design to incorporate this principle (Chen et al., 2021b; Garcia-Alonso et al., 2014). Second, it demonstrates the power of establishing species- and endpoint-specific natural variation ranges. These ranges offer a scientifically defensible “safe harbor” criterion: if a GE crop’s effects fall within this range, they can be considered ecologically negligible.

Our findings also help reconcile the conflicting results in the literature. Studies that reported adverse effects of *Bt* maize on *D. magna* often used only a single *Bt* line and a single non-*Bt* control, without accounting for natural background variability (Bøhn et al., 2008, 2010; Holderbaum et al., 2015). In contrast, studies that included multiple controls or used purified Cry proteins typically found no effects (Chen et al., 2018a; Mendelson et al., 2003; Raybould and Vlachos, 2011; Raybould et al., 2014). Our results support the latter body of evidence and suggest that previously reported negative effects may have been confounded by plant background differences, nutritional stress from suboptimal diets, or a combination of both. As noted by Raybould et al. (2014), high concentrations of protein test substances can cause non-toxic effects in *D. magna* that may be misinterpreted as toxicity.

For the specific *Bt* maize lines HY1LP and MY73LP, we conclude that exposure to leachates from their major plant tissues poses no significant risk to *D. magna* populations beyond the risks already present and accepted from the cultivation of diverse conventional maize. The effects observed are predominantly due to natural plant variation and not to the introduced Cry proteins.

### 4.5 Nutritional stress as a deliberate worst−case exposure condition

We acknowledge that using maize materials as the sole food source for *D. magn*a may induce nutritional stress, as evidenced by the consistently lower performance of animals fed maize materials compared to those fed green algae (Chen et al., 2021a). However, this design was intentional and serves two key purposes. First, it represents a realistic worst−case exposure scenario: in agricultural landscapes, *D. magna* in water bodies adjacent to maize fields may indeed encounter maize detritus as a major food source during certain periods (Rosi−Marshall et al., 2007; Tank et al., 2010). By forcing exposure to maize materials as the exclusive diet, we maximized the chance of detecting any potential adverse effects of the Cry proteins. Second, all treatment groups, including *Bt* lines, near−isogenic controls, and the ten conventional varieties, were subjected to the identical level of nutritional stress. Consequently, the observed differences among maize lines cannot be attributed to the stress itself but rather to inherent genetic or compositional differences among the lines (i.e., plant background effects). Importantly, nutritional stress may amplify underlying differences among conventional maize lines, which, if anything, would make it more difficult to conclude that *Bt* lines pose no additional risk. Despite this amplification, all but three parameter means from *Bt* treatments fell within the natural variation range (VR) defined by the ten conventional varieties, and no consistent *Bt*−specific effects were detected. Thus, the intentional nutritional stress does not weaken our conclusions; rather, it provides a conservative testing framework that strengthens the evidence for negligible risk of the tested *Bt* maize lines.

### 4.6 Recommendations for future studies

Based on the findings and limitations of this study, we recommend that future non-target organism studies: (1) include multiple conventional varieties to establish a baseline of natural variation, as demonstrated in our previous work (Chen et al., 2021a; b) and further validated in this study; (2) when possible, include *Bt* events in multiple genetic backgrounds to separate background effects from transgene effects, following the approach of Jensen et al. (2010) and Swan et al. (2009); and (3) carefully consider the nutritional adequacy of test materials to avoid confounding due to dietary stress (Chen et al., 2021a). Additionally, future work should examine effects under more environmentally realistic settings (e.g., outdoor mesocosms) and apply the VR framework to other aquatic invertebrates. Metabolomic profiling may also help identify specific phytochemicals driving plant background effects.

## 5 Conclusions

This study provides a robust framework for improving the environmental risk assessment of GE crops. By integrating a multi-tissue exposure design with a diverse panel of conventional varieties to establish a natural variation range, we successfully disentangled plant background effects from *Bt* protein effects. Our results demonstrate that the two *Bt* maize lines tested, despite expressing multiple Cry proteins at varying concentrations across tissues, do not cause consistent or biologically significant adverse effects on *D. magna* beyond the natural variability inherent in conventional maize. Applying this framework, we conclude that under the conditions tested the *Bt* maize lines tested in this study pose negligible risk to *D. magna* under realistic worst-case exposure scenarios.

## Data Availability

The original contributions presented in the study are included in the article/**Supplementary Material**. Further inquiries can be directed to the corresponding author.

## Supplementary material

Supplementary Table 1 Medium quality parameters [pH value; dissolved oxygen concentration (DOC)] of ADAM medium containing maize materials (pollen, leaves, flour) or straw soaking solution containing algae (*Chlorella pyrenoidosa*) from twelve maize lines (including ten conventional maize lines and two transgenic *Bt* maize lines). Values represent means ± SE.
