# Supplementary material for: Plant background, not Bt proteins, drives Daphnia magna life table responses: a multi-tissue chronic study with multi-Cry Bt maize
Source: Front Plant Sci. 2026 Jun 23;17:1852457. doi: 10.3389/fpls.2026.1852457 (PMC13337643; doi:10.3389/fpls.2026.1852457)
Supplement: Supplementary Table 1 — Medium quality parameters [pH value; dissolved oxygen concentration (DOC)] of ADAM medium containing maize materials (pollen, leaves, flour) or straw soaking solution containing algae (Chlorella pyrenoidosa) from twelve maize lines (including ten conventional maize lines and two transgenic Bt maize lines). Values represent means ± SE. [file Table1.docx]

Supplementary Material

# **Supplemental Information**

This supplementary information provides detailed descriptions of the 12 maize lines used in the main experiment, as well as complete water quality data monitored throughout the 21 day chronic toxicity test (Table S1).

The eight conventional maize varieties, two transgenic *Bt* maize lines and the corresponding non-*Bt* maize lines were:

Lianchuang 839 (LC839), exhibits moderate resistance to northern corn leaf blight and head smut, high resistance to Fusarium stalk rot, resistance to Fusarium ear rot, and susceptibility to gray leaf spot;

Ludan 9191 (LD9191), exhibits resistance to stalk rot and southern corn leaf blight, susceptibility to ear rot and Curvularia leaf spot, and high susceptibility to southern rust and common smut;

Longping 722 (LP722), resistant to northern corn leaf blight (3R), susceptible to Curvularia leaf spot (7S) and head smut (16.0%S), moderately resistant to stalk rot (16.7%MR), and susceptible to corn borer (6.1S);

Longtian 5173 (LT5173), susceptible to head smut, highly resistant to stalk rot, resistant to ear rot, moderately resistant to northern corn leaf blight, and susceptible to maize dwarf mosaic;

Longyuan 916 (LY916), susceptible to northern corn leaf blight and gray leaf spot, and moderately resistant to stalk rot, ear rot, and head smut;

Liangyu 99 (LY99), resistant to Curvularia leaf spot, moderately resistant to northern corn leaf blight, head smut, and stalk rot, and has strong lodging resistance;

Qinfeng 108 (QF108), susceptible to head smut and maize dwarf mosaic, moderately resistant to stalk rot and northern corn leaf blight, and resistant to ear rot;

Yudan 517 (YD517), resistant to Fusarium ear rot and common smut, moderately resistant to Fusarium stalk rot, Curvularia leaf spot, and southern rust, and susceptible to southern corn leaf blight;

Hengyu 1 (HY1), susceptible to northern corn leaf blight (7S) and Curvularia leaf spot (7S), moderately resistant to head smut (7.1%MR) and corn borer (5.1MR), and resistant to stalk rot (8.8%R);

MY73, resistant to stalk rot, moderately resistant to southern corn leaf blight, Curvularia leaf spot, common smut, and southern rust, and susceptible to ear rot;

HY1LP, expressing cry1Ab, cry2Ab, cry1F, with a genetic background HY1;

MY73LP, expressing the same cry1Ab, cry2Ab, cry1F, with a genetic background MY73.

**Table S1.** Medium quality parameters (pH value; dissolved oxygen concentration (DOC)) of ADAM medium containing maize materials (pollen, leaves, flour) or straw soaking solution containing algae (*Chlorella pyrenoidosa*) from twelve maize lines (including ten conventional maize lines and two transgenic *Bt* maize lines). Values represent means ± SE.

| **Treatment** | **Variety** | **pH vaule** | | | | | | | **DOC (mg/L)** | | | | | | |
| --- | --- | --- | --- | --- | --- | --- | --- | --- | --- | --- | --- | --- | --- | --- | --- |
|  |  | W0^a^ | W1^b^ | W2^c^ | W3^d^ | W4^e^ | W5^f^ | W6^g^ | W0^a^ | W1^b^ | W2^c^ | W3^d^ | W4^e^ | W5^f^ | W6^g^ |
| Pollen | LC839 | 7.34±0.030 | 7.34±0.034 | 7.41±0.029 | 7.35±0.047 | 7.18±0.037 | 7.08±0.037 | 7.21±0.039 | 7.98±0.041 | 7.91±0.019 | 8.76±0.024 | 8.70±0.040 | 8.65±0.062 | 8.59±0.097 | 8.88±0.162 |
|  | LD9191 |  | 7.43±0.050 | 7.23±0.057 | 7.33±0.020 | 7.18±0.043 | 7.19±0.053 | 7.23±0.058 |  | 7.84±0.009 | 8.78±0.058 | 8.70±0.095 | 8.82±0.081 | 8.67±0.036 | 8.71±0.121 |
|  | LP722 |  | 7.35±0.035 | 7.14±0.045 | 7.28±0.046 | 7.31±0.023 | 7.36±0.032 | 7.21±0.049 |  | 7.57±0.039 | 8.66±0.012 | 8.52±0.024 | 8.53±0.131 | 8.50±0.226 | 8.71±0.025 |
|  | LT5173 |  | 7.71±0.061 | 7.26±0.019 | 7.16±0.020 | 7.17±0.039 | 7.08±0.015 | 7.18±0.029 |  | 8.23±0.038 | 8.27±0.141 | 9.02±0.040 | 8.73±0.103 | 8.13±0.048 | 8.57±0.075 |
|  | LY916 |  | 7.53±0.032 | 7.28±0.049 | 7.16±0.050 | 7.14±0.032 | 7.14±0.021 | 7.18±0.034 |  | 8.25±0.035 | 8.27±0.017 | 9.03±0.052 | 8.87±0.107 | 8.49±0.180 | 8.29±0.264 |
|  | LY99 |  | 7.35±0.034 | 7.19±0.012 | 7.18±0.038 | 7.19±0.036 | 7.15±0.040 | 7.24±0.038 |  | 7.54±0.071 | 8.74±0.017 | 8.55±0.034 | 8.93±0.021 | 8.70±0.075 | 8.67±0.118 |
|  | QF108 |  | 7.43±0.023 | 7.13±0.023 | 7.15±0.043 | 7.09±0.035 | 7.11±0.007 | 7.16±0.035 |  | 7.91±0.043 | 8.82±0.072 | 8.63±0.121 | 8.59±0.097 | 8.63±0.126 | 8.89±0.019 |
|  | YD517 |  | 7.32±0.024 | 7.28±0.029 | 7.30±0.046 | 7.15±0.038 | 7.14±0.049 | 7.19±0.028 |  | 7.91±0.040 | 8.81±0.071 | 8.71±0.055 | 8.53±0.068 | 8.56±0.049 | 8.77±0.015 |
|  | HY1 |  | 7.52±0.028 | 7.35±0.062 | 7.12±0.012 | 7.13±0.032 | 7.14±0.034 | 7.19±0.035 |  | 8.21±0.045 | 8.30±0.017 | 8.95±0.034 | 8.30±0.048 | 7.86±0.054 | 8.37±0.042 |
|  | MY73 |  | 7.45±0.068 | 7.32±0.026 | 7.12±0.028 | 7.19±0.015 | 7.14±0.023 | 7.20±0.032 |  | 8.29±0.037 | 8.24±0.043 | 9.00±0.055 | 8.32±0.096 | 7.82±0.096 | 8.19±0.081 |
|  | HY1LP |  | 7.28±0.067 | 7.33±0.023 | 7.31±0.048 | 7.35±0.017 | 7.41±0.064 | 7.15±0.035 |  | 7.53±0.063 | 8.47±0.031 | 8.27±0.083 | 8.53±0.035 | 8.33±0.090 | 8.35±0.052 |
|  | MY73LP |  | 7.39±0.022 | 7.28±0.035 | 7.36±0.023 | 7.44±0.017 | 7.38±0.019 | 7.19±0.041 |  | 7.44±0.050 | 8.42±0.049 | 8.39±0.098 | 8.32±0.050 | 8.19±0.052 | 8.25±0.055 |
| Leaves | LC839 |  | 7.43±0.032 | 7.28±0.012 | 7.50±0.038 | 7.25±0.018 | 7.21±0.032 | 7.27±0.024 |  | 8.09±0.058 | 9.04±0.040 | 8.87±0.072 | 8.59±0.022 | 8.31±0.108 | 8.66±0.038 |
|  | LD9191 |  | 7.64±0.017 | 7.58±0.030 | 7.52±0.055 | 7.58±0.027 | 7.54±0.031 | 7.69±0.038 |  | 7.72±0.055 | 8.88±0.019 | 8.78±0.073 | 8.70±0.067 | 8.26±0.091 | 8.42±0.087 |
|  | LP722 |  | 7.36±0.021 | 7.56±0.039 | 7.51±0.013 | 7.49±0.044 | 7.36±0.019 | 7.52±0.055 |  | 8.11±0.018 | 9.07±0.093 | 8.65±0.035 | 8.40±0.006 | 8.34±0.084 | 8.66±0.067 |
|  | LT5173 |  | 7.60±0.042 | 7.72±0.036 | 7.78±0.042 | 7.56±0.006 | 7.58±0.013 | 7.72±0.020 |  | 7.79±0.047 | 8.53±0.024 | 8.35±0.083 | 8.45±0.013 | 8.37±0.090 | 8.16±0.067 |
|  | LY916 |  | 7.43±0.032 | 7.39±0.009 | 7.57±0.056 | 7.36±0.031 | 7.38±0.057 | 7.52±0.033 |  | 8.16±0.050 | 9.11±0.070 | 8.77±0.055 | 8.54±0.041 | 8.08±0.058 | 8.74±0.094 |
|  | LY99 |  | 7.60±0.017 | 7.57±0.030 | 7.49±0.020 | 7.56±0.025 | 7.54±0.009 | 7.66±0.012 |  | 7.68±0.057 | 8.40±0.108 | 8.33±0.049 | 8.61±0.015 | 8.30±0.019 | 8.39±0.053 |
|  | QF108 |  | 7.38±0.012 | 7.45±0.026 | 7.51±0.007 | 7.26±0.034 | 7.15±0.028 | 7.36±0.058 |  | 8.28±0.012 | 9.13±0.070 | 8.98±0.037 | 8.84±0.047 | 8.09±0.140 | 8.85±0.058 |
|  | YD517 |  | 7.58±0.007 | 7.57±0.013 | 7.56±0.006 | 7.59±0.015 | 7.46±0.018 | 7.63±0.006 |  | 7.70±0.027 | 8.40±0.027 | 8.37±0.026 | 8.49±0.038 | 8.22±0.070 | 8.35±0.076 |
|  | HY1 |  | 7.43±0.026 | 7.50±0.028 | 7.40±0.049 | 7.35±0.028 | 7.36±0.034 | 7.49±0.030 |  | 8.23±0.033 | 9.05±0.054 | 8.93±0.025 | 8.61±0.083 | 8.26±0.062 | 8.61±0.033 |
|  | MY73 |  | 7.55±0.013 | 7.52±0.003 | 7.45±0.012 | 7.56±0.023 | 7.50±0.015 | 7.60±0.020 |  | 7.68±0.025 | 8.26±0.076 | 7.90±0.017 | 8.50±0.045 | 8.34±0.114 | 8.45±0.015 |
|  | HY1LP |  | 7.42±0.023 | 7.61±0.026 | 7.51±0.017 | 7.32±0.028 | 7.19±0.038 | 7.33±0.048 |  | 7.83±0.003 | 9.02±0.009 | 8.94±0.024 | 8.39±0.075 | 8.26±0.038 | 8.56±0.032 |
|  | MY73LP |  | 7.63±0.021 | 7.60±0.010 | 7.53±0.015 | 7.59±0.032 | 7.57±0.013 | 7.81±0.007 |  | 7.68±0.047 | 8.94±0.006 | 8.54±0.038 | 8.67±0.053 | 8.17±0.013 | 8.42±0.029 |
| Flour | LC839 |  | 7.52±0.025 | 7.22±0.020 | 7.18±0.030 | 7.14±0.018 | 7.14±0.032 | 7.11±0.007 |  | 8.36±0.090 | 8.66±0.053 | 8.69±0.060 | 8.64±0.072 | 8.49±0.090 | 8.39±0.043 |
|  | LD9191 |  | 7.22±0.050 | 7.14±0.010 | 7.06±0.017 | 7.09±0.038 | 7.07±0.044 | 7.04±0.018 |  | 8.19±0.078 | 8.96±0.100 | 8.90±0.087 | 8.89±0.030 | 8.75±0.023 | 8.75±0.058 |
|  | LP722 |  | 7.79±0.017 | 7.28±0.032 | 7.04±0.015 | 7.10±0.009 | 7.22±0.015 | 7.24±0.031 |  | 7.94±0.031 | 8.88±0.035 | 9.03±0.022 | 8.90±0.080 | 8.51±0.118 | 8.62±0.044 |
|  | LT5173 |  | 7.61±0.098 | 7.35±0.247 | 7.10±0.027 | 7.09±0.015 | 7.02±0.009 | 7.17±0.049 |  | 7.97±0.031 | 8.93±0.055 | 8.94±0.061 | 9.00±0.027 | 8.46±0.045 | 8.60±0.019 |
|  | LY916 |  | 7.79±0.052 | 7.24±0.025 | 7.23±0.042 | 7.24±0.003 | 7.12±0.052 | 7.23±0.035 |  | 7.99±0.023 | 8.96±0.032 | 8.94±0.036 | 8.67±0.043 | 8.29±0.054 | 8.46±0.049 |
|  | LY99 |  | 7.36±0.107 | 7.36±0.030 | 7.09±0.023 | 7.36±0.040 | 7.10±0.010 | 7.22±0.013 |  | 7.89±0.060 | 8.74±0.090 | 8.80±0.120 | 8.68±0.043 | 8.32±0.007 | 8.33±0.037 |
|  | QF108 |  | 7.82±0.035 | 7.31±0.030 | 7.24±0.033 | 7.22±0.009 | 7.19±0.065 | 7.21±0.032 |  | 7.93±0.003 | 8.89±0.052 | 8.80±0.064 | 8.77±0.064 | 8.37±0.085 | 8.38±0.040 |
|  | YD517 |  | 7.08±0.029 | 7.04±0.017 | 7.03±0.020 | 7.06±0.021 | 7.03±0.003 | 7.06±0.026 |  | 8.09±0.035 | 8.92±0.044 | 8.92±0.006 | 8.83±0.035 | 8.68±0.034 | 8.88±0.048 |
|  | HY1 |  | 7.35±0.019 | 7.14±0.018 | 7.08±0.007 | 7.21±0.017 | 7.08±0.021 | 7.06±0.009 |  | 8.26±0.057 | 8.77±0.010 | 8.82±0.029 | 8.70±0.044 | 8.57±0.022 | 8.72±0.079 |
|  | MY73 |  | 7.41±0.023 | 7.19±0.021 | 7.10±0.012 | 7.20±0.003 | 7.12±0.030 | 7.11±0.046 |  | 8.29±0.069 | 8.70±0.021 | 8.80±0.096 | 8.58±0.055 | 8.38±0.057 | 8.36±0.105 |
|  | HY1LP |  | 7.65±0.037 | 7.35±0.009 | 7.21±0.035 | 7.28±0.015 | 7.10±0.009 | 7.11±0.019 |  | 7.96±0.045 | 8.93±0.022 | 8.96±0.044 | 8.82±0.109 | 8.52±0.033 | 8.45±0.115 |
|  | MY73LP |  | 7.48±0.024 | 7.20±0.007 | 7.14±0.038 | 7.15±0.015 | 7.11±0.015 | 7.13±0.007 |  | 8.38±0.035 | 8.74±0.052 | 8.78±0.086 | 8.58±0.049 | 8.43±0.012 | 8.15±0.070 |
| Straw | LC839 |  | 7.10±0.054 | 7.10±0.031 | 7.13±0.015 | 7.24±0.026 | 7.36±0.032 | 7.23±0.026 |  | 6.74±0.055 | 6.91±0.015 | 7.25±0.319 | 7.13±0.063 | 7.17±0.101 | 7.05±0.043 |
|  | LD9191 |  | 7.22±0.023 | 7.27±0.038 | 7.21±0.041 | 7.30±0.058 | 7.37±0.017 | 7.22±0.024 |  | 7.58±0.095 | 7.18±0.035 | 7.13±0.101 | 7.22±0.044 | 7.20±0.012 | 7.23±0.032 |
|  | LP722 |  | 7.18±0.057 | 7.13±0.087 | 7.20±0.059 | 7.24±0.114 | 7.32±0.069 | 7.25±0.080 |  | 7.47±0.119 | 7.34±0.045 | 7.23±0.028 | 7.22±0.084 | 7.22±0.046 | 7.20±0.035 |
|  | LT5173 |  | 7.24±0.023 | 7.27±0.024 | 7.21±0.052 | 7.41±0.033 | 7.40±0.064 | 7.30±0.078 |  | 7.45±0.056 | 7.34±0.049 | 7.37±0.124 | 7.34±0.086 | 7.40±0.092 | 7.31±0.026 |
|  | LY916 |  | 7.21±0.020 | 7.29±0.086 | 7.28±0.009 | 7.39±0.042 | 7.43±0.006 | 7.28±0.038 |  | 7.35±0.038 | 7.25±0.023 | 7.35±0.045 | 7.46±0.101 | 7.34±0.019 | 7.30±0.123 |
|  | LY99 |  | 7.28±0.007 | 7.23±0.098 | 7.26±0.046 | 7.34±0.087 | 7.38±0.032 | 7.16±0.095 |  | 7.69±0.211 | 7.34±0.095 | 7.16±0.056 | 7.24±0.032 | 7.25±0.033 | 7.45±0.094 |
|  | QF108 |  | 7.18±0.054 | 7.15±0.086 | 7.31±0.010 | 7.32±0.077 | 7.43±0.003 | 7.16±0.038 |  | 7.43±0.123 | 7.32±0.126 | 7.19±0.069 | 7.21±0.063 | 7.27±0.089 | 7.26±0.057 |
|  | YD517 |  | 7.04±0.030 | 7.23±0.021 | 7.27±0.054 | 7.34±0.050 | 7.34±0.028 | 7.23±0.023 |  | 7.42±0.057 | 7.27±0.061 | 7.15±0.085 | 7.39±0.023 | 7.34±0.025 | 7.39±0.039 |
|  | HY1 |  | 7.16±0.023 | 7.35±0.014 | 7.32±0.063 | 7.39±0.043 | 7.35±0.050 | 7.53±0.038 |  | 7.17±0.018 | 8.87±0.052 | 8.74±0.071 | 8.51±0.074 | 8.03±0.044 | 8.55±0.044 |
|  | MY73 |  | 7.14±0.019 | 7.44±0.030 | 7.39±0.057 | 7.54±0.038 | 7.64±0.012 | 7.48±0.052 |  | 7.17±0.015 | 8.98±0.080 | 8.57±0.043 | 8.51±0.035 | 7.84±0.030 | 8.55±0.012 |
|  | HY1LP |  | 7.12±0.022 | 7.44±0.047 | 7.30±0.012 | 7.35±0.087 | 7.41±0.090 | 7.27±0.030 |  | 6.64±0.043 | 8.82±0.118 | 8.59±0.047 | 8.05±0.071 | 6.76±0.075 | 7.89±0.107 |
|  | MY73LP |  | 7.28±0.058 | 7.33±0.100 | 7.31±0.049 | 7.33±0.112 | 7.41±0.040 | 7.43±0.082 |  | 7.85±0.041 | 8.95±0.030 | 8.65±0.058 | 8.45±0.077 | 7.90±0.046 | 8.74±0.030 |

^a^ W0: pure ADAM medium, n= 3.

^b^ W1: pure ADAM medium containing food, n=3.

^c^ W2: W1 after 24 hours, n=3.

^d^ W3: W2 with added food, n=3.

^e^ W4: W3 after 24 hours, n=3.

^f^ W5: W4 with added food, n=3.

^g^ W6: W5 after 24 hours, n=3.


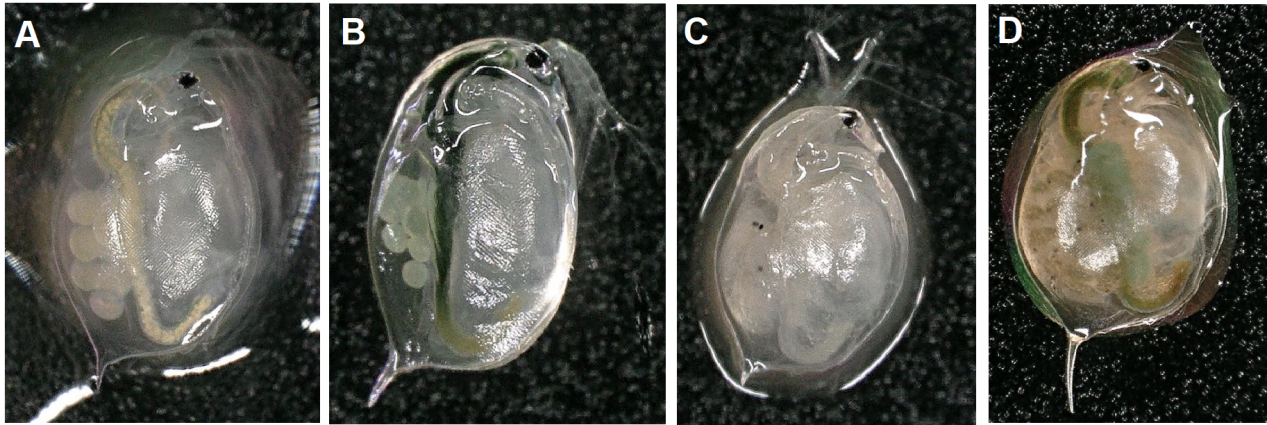


**Fig. S1.** Photographs of *D. magna* after feeding on A) maize pollen, B) maize leaves, C) maize flour, or D) maize straw soaking solution containing algae. Note the different color of the gut for the different maize materials.
